# Reduction in Pigment Epithelial Detachment Thickness with Faricimab versus Aflibercept 2 mg during Head-to-Head Dosing in TENAYA/LUCERNE

**DOI:** 10.1016/j.xops.2026.101148

**Published:** 2026-03-10

**Authors:** Jennifer I. Lim, Aude Ambresin, Robert L. Avery, Voraporn Chaikitmongkol, Nicole Eter, Fumi Gomi, Arshad M. Khanani, Nikolas J.S. London, Emma Harrell, Philippe Margaron, Shriji Patel, Audrey Souverain, Ming Yang, Timothy Y.Y. Lai

**Affiliations:** 1 University of Illinois at Chicago, Chicago, Illinois; 2 Swiss Visio Montchoisi, Lausanne, Switzerland; 3 California Retina Consultants, Santa Barbara, California; 4 Retina Division, Department of Ophthalmology, Faculty of Medicine, Chiang Mai University, Chiang Mai, Thailand; 5 University of Münster Medical Center, Münster, Germany; 6 Hyogo Medical University, Nishinomiya, Japan; 7 Sierra Eye Associates, Reno, Nevada; 8 University of Nevada, Reno School of Medicine, Reno, Nevada; 9 Retina Consultants San Diego, San Diego, California; 10 Roche Products Ltd, Welwyn Garden City, United Kingdom; 11 F. Hoffmann-La Roche Ltd., Basel, Switzerland; 12 Genentech, Inc., South San Francisco, California; 13 The Chinese University of Hong Kong, New Territories, Hong Kong

**Keywords:** Angiopoietin-2, Faricimab, Neovascular age-related macular degeneration, Pigment epithelial detachment, Retinal pigment epithelium

## Abstract

**Purpose:**

To evaluate the effects of dual angiopoietin-2 (Ang-2)/VEGF-A pathway inhibition with faricimab versus VEGF pathway inhibition with aflibercept 2 mg on pigment epithelial detachment (PED) in patients with neovascular age-related macular degeneration (nAMD).

**Design:**

TENAYA/LUCERNE (NCT03823287/NCT03823300) post hoc analysis.

**Participants:**

Patients with treatment-naïve nAMD.

**Methods:**

Patients were randomized 1:1 to faricimab 6 mg up to every 16 weeks (n = 665) after 4 initial every-4-week (Q4W) doses or aflibercept 2 mg every 8 weeks (n = 664) after 3 Q4W doses. Pigment epithelial detachment was defined as retinal pigment epithelium (RPE) elevation width ≥350 μm and graded as predominantly/purely serous (serous PED) or predominantly/only fibrovascular (fibrovascular PED). Large PED definition: thickness ≥125 μm.

**Main Outcome Measures:**

Pigment epithelial detachment thickness change from baseline during initial 12-week head-to-head dosing, proportion of patients with serous PED at the end of head-to-head dosing, and time to first reduction of maximum PED thickness by 50%.

**Results:**

Baseline PED characteristics were similar between arms. At week 12, the adjusted mean decrease from baseline in maximum PED thickness was greater with faricimab than aflibercept 2 mg in eyes with large (–119.1 [n = 500] vs. –101.4 μm [n = 496]; nominal *P* = 0.0028), serous (–136.1 [n = 128] vs. –108.2 μm [n = 114]; nominal *P* = 0.0147), and any type (–87.9 [n = 644] vs. –74.5 μm [n = 638]; nominal *P* = 0.0067) PED at baseline. The proportion of eyes with serous PED at baseline remaining serous at week 12 was lower with faricimab than aflibercept 2 mg (4.7% vs. 13.4%; nominal *P* = 0.0258). In eyes with large PED at baseline, the cumulative incidence of PEDs achieving time to first reduction of maximum PED thickness by 50% at week 12 was 35.3% with faricimab versus 25.7% with aflibercept 2 mg. The corresponding incidence in eyes with serous PED at baseline was 61.1% with faricimab versus 51.8% with aflibercept 2 mg. The incidence of RPE tears was low (faricimab, 2.9%; aflibercept 2 mg, 1.5%).

**Conclusions:**

In TENAYA/LUCERNE, dual Ang-2/VEGF-A inhibition with faricimab elicited greater improvements in PED outcomes versus aflibercept 2 mg during head-to-head dosing. These findings are consistent with the greater drying of retinal fluid with faricimab during head-to-head dosing, which may allow for rapid treatment interval extension.

**Financial Disclosure(s):**

Proprietary or commercial disclosure may be found in the Footnotes and Disclosures at the end of this article.

Pigment epithelial detachment (PED) is a relatively common clinical feature in patients with neovascular age-related macular degeneration (nAMD), which occurs when the retinal pigment epithelium (RPE) detaches from the Bruch membrane due to a buildup of fluid, fibrovascular membrane, drusenoid material, or blood.^1^ The reported incidence of PED in nAMD ranges from 63% to >90%,^2^, ^3^, ^4^, ^5^, ^6^, ^7^ with this variability likely reflecting different definitions used and different patient populations evaluated across studies. For instance, there is a high PED incidence (94%) in patients with vitreous hemorrhage secondary to polypoidal choroidal vasculopathy (94%)^7^ and in patients with retinal angiomatous proliferation (86%).^8^ In nAMD, PED is categorized on OCT as serous, fibrovascular, or mixed type serous and fibrovascular.^1^ Serous PEDs appear as dome-shaped RPE elevations with underlying hyporeflective fluid and distinct borders,^1^^,^^9^^,^^10^ whereas fibrovascular PEDs are filled with a combination of hyperreflective and hyporeflective fluid.^9^ Due to the chronic and slow progression of serous PEDs, patients are typically not at immediate risk for vision loss.^1^ In contrast, patients with newly diagnosed fibrovascular PEDs typically experience significant vision loss (>3 lines) due to consequences of the choroidal neovascular membrane in the absence of treatment.^1^

Central subfield thickness (CST), intraretinal fluid (IRF), and subretinal fluid (SRF) are established biomarkers for predicting outcomes in nAMD and are widely used to inform treatment decisions.^11^, ^12^, ^13^ To date, PED has not routinely been included in the decision-making process for nAMD treatment^13^ but is garnering interest as a potential predictor of treatment response.^14^^,^^15^ The presence of PED may indicate growth of neovascularization with fluid leakage, which can lead to poor visual function.^16^ Further, a number of studies have shown that the presence of PEDs is associated with vision loss,^14^^,^^17^ supporting PED as a biomarker of disease activity in nAMD.

Anti-VEGF therapy is effective in resolving IRF and SRF associated with PEDs.^3^ After treatment with anti-VEGF therapy, SRF and IRF resolve rapidly in ≥70% of eyes.^3^ In contrast, PED in eyes with nAMD is more recalcitrant to anti-VEGF therapy and PED resolution occurs more slowly.^3^ Similarly, although anti-VEGF therapy has also been demonstrated to reduce PED thickness in polypoidal choroidal vasculopathy, with posttreatment OCT evaluation of PED proposed to be a biomarker of response,^18^ some patients with polypoidal choroidal vasculopathy experience disease progression, despite ongoing anti-VEGF treatment.^3^^,^^19^ Given that PED has been reported to be a primary indicator of disease progression,^3^ treatments that offer improved resolution of PEDs over anti-VEGF therapy may lead to better outcomes.

Faricimab is a novel humanized bispecific immunoglobulin G monoclonal antibody designed for intraocular use that independently binds and inhibits angiopoietin-2 (Ang-2) and VEGF-A.^20^^,^^21^ Given the involvement of Ang-2 in mediating vascular homeostasis, permeability, and angiogenesis and sensitizing retinal vessels to VEGF-A,^20^^,^^22^^,^^23^ targeting the VEGF-A and Ang-2 pathways with faricimab may improve vascular stability and disease control, thereby increasing treatment durability. The TENAYA and LUCERNE trials were identical phase III randomized controlled trials designed to evaluate the efficacy, safety, and durability of faricimab 6.0 mg versus aflibercept 2.0 mg in treatment-naïve patients with nAMD over 2 years.^21^^,^^24^^,^^25^ Faricimab up to every 16 weeks improved vision and anatomy, with a high proportion of patients on extended treatment intervals (greater than or equal to every 12 weeks) based on protocol-defined disease activity criteria.^21^^,^^25^ Faricimab had a safety profile comparable with that of aflibercept 2.0 mg every 8 weeks (Q8W) through 2 years.^25^ In subsequent post hoc analyses, treatment with faricimab was found to result in a more pronounced reduction in CST and a greater proportion of patients achieving absence of both SRF and IRF versus aflibercept throughout head-to-head dosing (the first 12 weeks).^26^ Further, in patients with IRF and or SRF at baseline, first absence of IRF and SRF was achieved 4 weeks faster with faricimab during head-to-head dosing.^26^ Given these findings, we hypothesized that faricimab would also better improve PED versus aflibercept during head-to-head dosing.

The purpose of this post hoc analysis was to evaluate the effect of dual Ang-2/VEGF-A pathway inhibition with faricimab versus VEGF pathway inhibition with aflibercept on PED outcomes in TENAYA/LUCERNE. Specific PED outcomes evaluated included the change from baseline in PED thickness and the proportion of eyes with serous PED at end of head-to-head dosing. The time to first reduction of maximum PED thickness by 50% was also evaluated.

## Methods

### Study Design

This was a post hoc analysis of the pooled TENAYA (clinicaltrials.gov identifier: NCT03823287) and LUCERNE (clinicaltrials.gov identifier: NCT03823300) trials, the study design and rationale of which have been previously published.^21^^,^^24^

The study protocols were approved by appropriate regulatory authorities, applicable ethics committees, and institutional review boards. The 2 trials were conducted in accordance with the Declaration of Helsinki and followed principles of Good Clinical Practice. Written consent has been obtained from all patients.

### Participants

The full eligibility criteria for TENAYA and LUCERNE have been previously published.^21^ Briefly, these criteria included age ≥50 years; treatment-naïve choroidal neovascularization (CNV) secondary to nAMD, with subfoveal component related to CNV activity on OCT, and or CNV exudation on fundus fluorescein angiography; CNV lesion size ≤9 disc-areas and CNV component area of ≥50% of total lesion area on fundus fluorescein angiography; and best-corrected visual acuity (BCVA) of 78–24 ETDRS letters (20/32–20/320 approximate Snellen equivalent). There were no exclusion criteria for PED size.

### Treatment Protocol

Dosing procedures in TENAYA and LUCERNE have been previously described in detail.^21^^,^^24^^,^^25^

Patients were randomized 1:1 to receive intravitreal faricimab 6.0 mg up to every 16 weeks or aflibercept 2.0 mg Q8W. Patients initially received 4 doses of faricimab administered every 4 weeks (Q4W) or 3 doses of aflibercept Q4W. The head-to-head dosing phase was defined as the first 8 weeks of the 12-week initial dosing phase, during which patients in both treatment arms received 3 Q4W treatments (at weeks 0, 4, and 8). Patients receiving faricimab underwent protocol-defined disease activity assessments at weeks 20 and 24, which determined whether they were assigned to faricimab Q8W, every 12 weeks, or every 16 weeks dosing intervals through week 60. Patients who received faricimab were then (after week 60) treated according to a treat-and-extend–based regimen. Patients in the aflibercept arm received aflibercept 2.0 mg Q8W after the initial 3 injections.

### PED Definition and Grading

Ocular images obtained throughout the study were independently assessed at baseline and at monthly follow-up visits by masked evaluators at the central reading centers, which harmonized their grading of the PED variables. Pigment epithelial detachment was defined as an elevation of the RPE on OCT with a width of ≥350 μm. Pigment epithelial detachment thickness (elevation in μm measured from the RPE to the underlying Bruch membrane layer) was the maximum thickness of the tallest PED within the 6-mm ETDRS grid. Pigment epithelial detachments with a maximum thickness of ≥125 μm were defined as large PEDs. The cut-off for “large” PED was selected as this was the lower quartile for maximum PED thickness of study eyes at baseline.

Pigment epithelial detachment subtype was determined by the central reading centers according to the relative amount of hyperreflectivity or hyporeflectivity of the PED closest to the foveal center. For the purpose of these analyses, fibrovascular PED was defined as eyes with predominantly fibrovascular PED or fibrovascular only PED and serous PED as eyes with predominantly serous PED or serous only PED.

### Outcomes

The adjusted mean PED thickness change from baseline was evaluated at weeks 4, 8, and 12 in study eyes with any, large, serous, and fibrovascular PED at baseline. The proportion of study eyes with serous PED at baseline that still had serous PED at week 12 was also evaluated. In addition, the time to first reduction of maximum PED thickness by 50% through week 60 was determined in eyes with large and serous PEDs at baseline.

The proportion of eyes with RPE tears was assessed through study end. Retinal pigment epithelium tear severity was defined by the investigators using standard adverse event severity grading as follows: mild = present but no disruption in normal daily activity; moderate = present and sufficient to reduce or affect normal daily activity; and severe = incapacitating with inability to work or perform normal daily activity.

### Statistical Analysis

Longitudinal analysis of PED thickness change from baseline was based on a mixed model for repeated measures analysis adjusted for treatment group, visit, visit-by-treatment group interaction, baseline PED (continuous), PED type at baseline (fibrovascular vs. serous), baseline BCVA (≥74, 73–55, and ≤54 letters), baseline low-luminance deficit (<33 and ≥33 letters), region (United States and Canada, Asia, and the rest of the world), reading center (Vienna vs. Duke), and trial (TENAYA vs. LUCERNE). Intercurrent events were handled following the same strategies used in the primary analysis.^21^^,^^24^ Sensitivity analyses were conducted where data from patients with RPE tears were censored from the time of RPE tear occurrence. Missing data were implicitly imputed by the model.

The time to first reduction of maximum PED thickness by 50% through week 60 was analyzed using Kaplan–Meier estimates; *P* values were generated from the stratified log-rank test. Hazard ratios were computed using the Cox model. The log-rank test and the Cox model were adjusted for baseline PED type (fibrovascular vs. serous), baseline BCVA (≥74, 73–55, and ≤54 letters), baseline low-luminance deficit (<33 and ≥ 33 letters), region (United States and Canada, Asia, and the rest of the world), reading center (Vienna vs. Duke), and trial (TENAYA vs. LUCERNE). Treatment policy strategy and hypothetical strategy were applied to non–coronavirus disease 2019-related and coronavirus disease 2019–related intercurrent events, respectively. Missing data were not imputed.

The proportion of eyes with serous PED at baseline that still had serous PED at week 12 was analyzed using Cochran–Mantel–Haenszel (CMH)-weighted estimates, which were based on a CMH test stratified by baseline BCVA (≥74, 73–55, and ≤54 letters), baseline low-luminance deficit (<33 and ≥33 letters), region (United States and Canada, Asia, and the rest of the world), and trial (TENAYA vs. LUCERNE). The nominal *P* value was obtained using the CMH test for superiority. Intercurrent events were handled following the same strategies used in the primary analysis.^21^^,^^24^ Sensitivity analyses were conducted where data from patients with RPE tears were censored from the time of RPE tear occurrence. Missing data were not imputed.

All *P* values reported in this manuscript are nominal and were not adjusted for multiplicity.

Safety analysis was conducted on the safety population, which included all randomized patients who received ≥1 dose of faricimab or aflibercept, grouped according to actual treatment received.

## Results

### Baseline Demographics and Clinical Characteristics

The overall baseline demographics and characteristics have been previously described;^26^ baseline PED-related characteristics are summarized in Table 1, and baseline ocular characteristics for eyes with large (faricimab n = 500; aflibercept 2 mg n = 496), serous (faricimab n = 128; aflibercept 2 mg n = 114), and fibrovascular PED (faricimab n = 664; aflibercept 2 mg n = 638) are summarized in Table S2 (available at www.ophthalmologyscience.org). As expected due to the trial definition of PED, the majority of eyes in both treatment arms had a PED involving the foveal center at baseline (Table 1). Pigment epithelial detachment thickness at baseline was balanced between treatment arms and approximately one-quarter of eyes with PED at baseline had a shallow PED with a maximum PED thickness of <125 μm. Among eyes with PED at baseline, the proportion of serous versus fibrovascular PED was also balanced between arms, with approximately 20% graded as serous, and the remaining 80% graded as fibrovascular (Table 1).

**Table 1:** Baseline PED Characteristics of Patients in TENAYA and LUCERNE

Characteristic	Faricimab up to Q16W (N = 665)	Aflibercept Q8W (N = 664)
Presence of PED,∗ n (%)	n = 664	n = 663
PED in any location	647 (97.4)	638 (96.2)
PED in center 1 mm	604 (91.0)	606 (91.4)
PED at foveal center	486 (73.2)	469 (70.7)
Type of PED,∗ n (%)	647	638
Predominantly serous	125 (19.3)	94 (14.7)
Purely serous	5 (0.8)	20 (3.1)
Predominantly fibrovascular	265 (41.0)	290 (45.5)
Fibrovascular only	252 (38.9)	234 (36.7)
Maximum thickness of PED,† μm	n = 656	n = 660
Mean ± SD	252.9 ± 188.5	239.8 ± 185.9
Median (Q1–Q3)	185.0 (126.5–316.0)	176.5 (125.0–300.5)

PED = pigment epithelial detachment; Q = quartile; Q8W = every 8 weeks; Q16W = every 16 weeks; SD = standard deviation.

This table includes all patients randomized in the trials grouped according to the treatment assigned at randomization.

∗ Percentages are based on the number of patients with PED type nonmissing at baseline.

† As measured within the 6-mm ETDRS grid.

### Change from Baseline in Maximum PED Thickness

**Figure 1:**
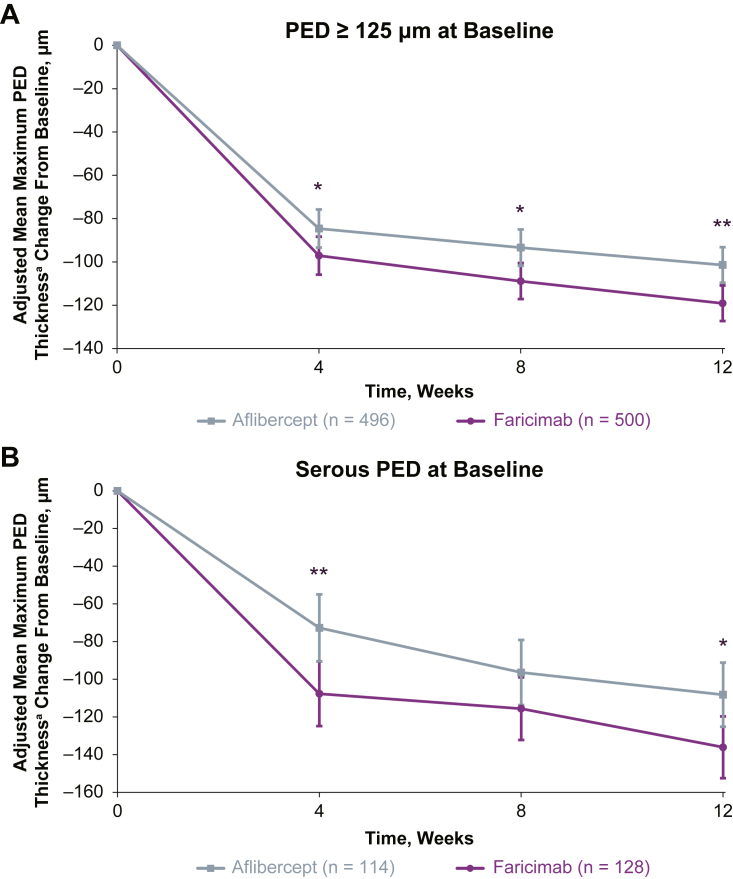
Reduction in maximum PED thickness with faricimab vs. aflibercept in the head-to-head dosing phase in patients with (**A**) PED ≥125 μm at baseline and (**B**) serous PED at baseline. ∗Nominal *P* < 0.05 vs. aflibercept; ∗∗Nominal *P* < 0.01 vs. aflibercept. *P* values are nominal and not adjusted for multiplicity; no formal statistical conclusion should be made based on the *P* values. ^a^Within the 6-mm ETDRS grid. Pigment epithelial detachment thickness results are based on a mixed model for repeated measures analysis on all patients randomized in the trials grouped according to the treatment assigned at randomization. The model adjusted for treatment group, visit, visit-by-treatment group interaction, baseline PED (continuous), PED type at baseline (fibrovascular vs. serous), baseline BCVA (≥74, 73–55, and ≤ 54 letters), baseline LLD (<33 and ≥ 33 letters), region (United States and Canada, Asia, and the rest of the world), reading center (Vienna vs. Duke), and study (TENAYA vs. LUCERNE). Treatment policy strategy and hypothetical strategy were applied to non–COVID-19–related and COVID-19–related intercurrent events, respectively. Ninety-five percent CIs are shown. Presence of PED defined as measured maximum thickness of PED within the 6-mm ETDRS grid at baseline. BCVA = best-corrected visual acuity; CI = confidence interval; COVID-19 = coronavirus disease 2019; LLD = low-luminance deficit; PED = pigment epithelial detachment.

In eyes with large PED at baseline, faricimab resulted in a greater decrease (nominal *P* < 0.05) in maximum PED thickness throughout head-to-head dosing compared with aflibercept (Fig 1A). At the end of head-to-head dosing (week 12), adjusted mean (95% confidence interval [CI]) changes from baseline in max PED thickness were –119.1 μm (–127.3, –110.9) with faricimab versus –101.4 μm (–109.6, –93.2) with aflibercept (mean difference vs. aflibercept [95% CI], –17.7 μm [–29.3, –6.1]; nominal *P* = 0.0028).

In eyes with serous PED at baseline, faricimab resulted in a greater decrease in maximum PED thickness throughout head-to-head dosing compared with aflibercept (Fig 1B), with nominally significant differences (nominal *P* < 0.05) at weeks 4 and 12. At week 12, adjusted mean (95% CI) changes from baseline in max PED thickness were –136.1 μm (–152.5, –119.7) with faricimab versus –108.2 μm (–125.2, –91.2) with aflibercept (mean difference vs. aflibercept [95% CI], –27.9 μm [–50.3, –5.5]; nominal *P* = 0.0147).

In eyes with any PED type at baseline, there was a comparable decrease in adjusted mean maximum PED thickness from baseline at week 4 between treatment arms (Fig S2A, available at www.ophthalmologyscience.org), with nominally significant differences (nominal *P* < 0.05) at weeks 8 and 12 that favored faricimab. At week 12, adjusted mean (95% CI) changes from baseline in maximum PED thickness were –87.9 μm (–94.8, –81.1) with faricimab versus –74.5 μm (–81.4, –67.6) with aflibercept (mean difference [95% CI], –13.5 μm [–23.2, –3.7]; nominal *P* = 0.0067).

In eyes with fibrovascular PED at baseline, corresponding adjusted mean changes from baseline were similar between treatment arms throughout head-to-head dosing (Fig S2B, available at www.ophthalmologyscience.org).

Results of the sensitivity analysis were consistent with the main analysis results.

The proportion of eyes with serous PED at baseline that were still serous at week 12 was lower with faricimab than aflibercept (4.7% vs. 13.4%; CMH weighted difference, –8.4% [95% CI: –14.7, –2.0]; nominal *P* = 0.0258; Fig S3, available at www.ophthalmologyscience.org).

### Time to First Reduction of Maximum PED Thickness by 50%

**Figure 4:**
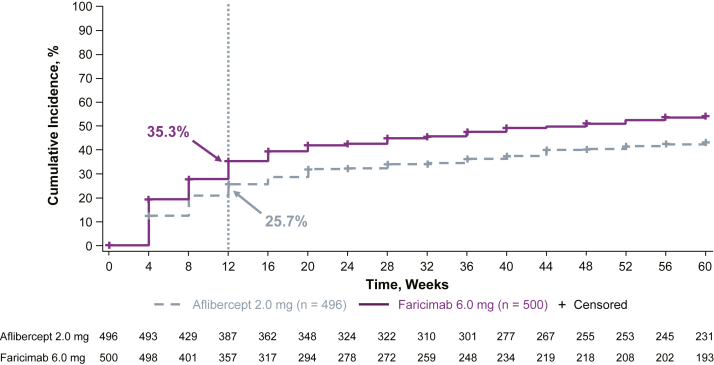
Time to first reduction in maximum PED thickness by 50% in patients with presence of PED ≥125 μm at baseline. Event is defined as first reduction in PED within the 6-mm ETDRS grid by 50% from baseline. Summaries of time to first reduction in PED within the 6-mm ETDRS grid by 50% from baseline are Kaplan–Meier estimates, with the time variable defined as the target visit week. Patients with missing PED at baseline were excluded from the analysis. PED = pigment epithelial detachment.

In eyes with large PED at baseline, the time to first reduction of maximum PED thickness by 50% was faster with faricimab than aflibercept (Fig 4). At week 12, the cumulative incidence (95% CI) of PEDs achieving time to first reduction of maximum PED thickness by 50% was 35.3% (31.0%–39.5%) with faricimab versus 25.7% (21.8%–29.5%) with aflibercept. The median (95% CI) was reached at week 48 (36–not evaluable) for faricimab and was not reached during the study period for aflibercept. The estimated hazard ratio (95% CI) was 1.57 (1.27–1.94; nominal *P* < 0.0001), meaning there was a 57% increase in the chance of 50% thickness reduction through week 60 in the faricimab arm compared with the aflibercept arm.

**Figure 5:**
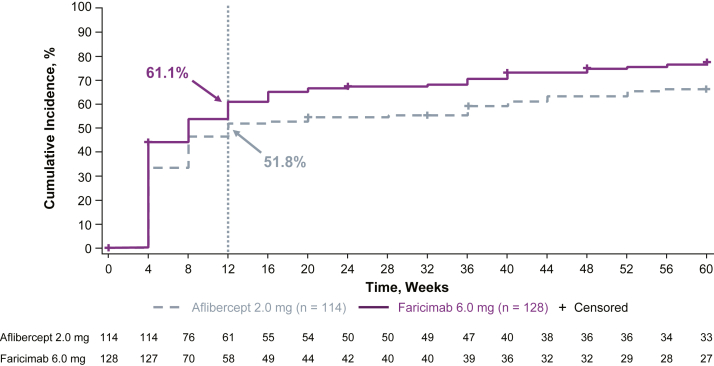
Time to first reduction in maximum PED thickness by 50% in patients with serous PED at baseline. Event is defined as first reduction in PED within the 6-mm ETDRS grid by 50% from baseline. Summaries of time to first reduction in PED within the 6-mm ETDRS grid by 50% from baseline are Kaplan–Meier estimates, with the time variable defined as the target visit week. Patients with missing PED at baseline were excluded from the analysis. PED = pigment epithelial detachment.

Similarly, in eyes with serous PED at baseline, the time to first reduction of maximum PED thickness by 50% was also faster with faricimab than aflibercept (Fig 5). At week 12, the cumulative incidence of time to first reduction of maximum PED thickness by 50% was 61.1% (52.6%–69.6%) with faricimab versus 51.8% (42.6%–60.9%) with aflibercept. The median (95% CI) was reached at week 8 (4–12) with faricimab and week 12 (8–36) with aflibercept. The 75th percentile was reached at week 52 with faricimab and was not reached with aflibercept during the study period. The estimated hazard ratio (95% CI) was 1.61 (1.05–2.47; nominal *P* = 0.0267), meaning there was a 61% increase in the chance of 50% thickness reduction through week 60 in the faricimab arm compared with the aflibercept arm.

### PED and BCVA

A treatment agnostic analysis revealed that, overall, the presence of PED and persistence of PED in the macula the first 12 weeks of treatment had a limited impact on functional outcomes. The presence of PED at the foveal center with a maximum PED thickness of ≥125 μm at baseline was associated with a trend toward worse functional outcomes (Fig S6, available at www.ophthalmologyscience.org). A similar trend was observed for PED presence at the foveal center with a maximum PED thickness of ≥125 μm at week 12 (Fig S7, available at www.ophthalmologyscience.org).

### RPE Tears

The incidence of RPE tears was low across both treatment arms (faricimab, 2.9% [19 eyes]; aflibercept, 1.5% [10 eyes]). The numerically higher incidence in the faricimab arm was not statistically significant compared with the aflibercept arm (difference in RPE frequency between arms: 1.50% [95% CI: –0.19, 3.30]). Among 4 patients with serious RPE tears in the faricimab arm, 2 had an associated BCVA loss of >15 letters at event onset, including 1 patient who experienced a BCVA loss of >30 letters.

Retinal pigment epithelium tears were associated with larger baseline PED thickness, and the majority occurred during the first 12 weeks of Q4W treatment. Approximately half of all eyes with an RPE tear had a fibrovascular PED at baseline (faricimab, 10/19 [52.6%]; aflibercept, 5/9 [55.6%]), and most had occult CNV at baseline (faricimab, 12/19 [63.2%]; aflibercept, 8/9 [88.9%]).

## Discussion

In this post hoc analysis of the TENAYA/LUCERNE trials, dual Ang-2/VEGF-A pathway inhibition with faricimab resulted in improved PED outcomes during head-to-head dosing compared with VEGF pathway inhibition alone with aflibercept. Specifically, these analyses showed that in patients with PED at baseline, and particularly in those exhibiting large or serous PED, greater reductions in PED thickness were seen with faricimab versus aflibercept at the end of the 12-week head-to-head dosing phase. There was no difference between the treatment arms in the change in PED thickness from baseline to week 12 in patients with fibrovascular PED at baseline. This may reflect fibrovascular PEDs being smaller than serous PEDs, on average, at baseline, making the detection of differences more challenging. We also found that the median time to first reduction of maximum PED thickness by 50% was shorter with faricimab than aflibercept in patients with large or serous PED at baseline. These results are consistent with findings demonstrating reductions in CST and retinal fluid (biomarkers of outcomes in nAMD) with faricimab versus aflibercept during head-to-head dosing in TENAYA and LUCERNE.^26^ Large and serous PEDs contain more sub-RPE fluid; however, regardless of whether more fluid was present in the retina or sub-RPE, a better response was seen with faricimab versus aflibercept.

Cross-trial comparison of PED data is complicated due to different definitions of PED. This post hoc analysis of TENAYA and LUCERNE focused on identifying PED with a width of ≥350 μm as per the definition used by the trial reading centers. Further, to focus on the treatment effect on PEDs of clinical significance, a subanalysis was performed, excluding eyes with shallow PED, by analyzing the ∼75% of eyes in this study with a maximum PED thickness at baseline of ≥125 μm. Other trials have defined PED differently, including a width of ≥400 μm or a thickness of ≥200 μm,^3^^,^^27^ and a width of 500 μm and a thickness of 100 μm.^28^ These definitions may have resulted in fewer RPE elevations being defined as PED compared with the definition used for TENAYA/LUCERNE.

These data provide the first insights into the effect of faricimab on PEDs in clinical trials; however, there is also growing body of evidence from clinical practice/real-world studies showing reductions in PED thickness and resolution of PED in patients with nAMD after switching from anti-VEGFs to faricimab.^29^, ^30^, ^31^, ^32^

The clinical relevance of our findings is supported by an increasing body of evidence indicating that PEDs may be a biomarker of disease activity in nAMD. For instance, in a treatment agnostic analysis of data from the HAWK/HARRIER phase III trials, Sarraf et al^14^ demonstrated that there is an inverse relationship between PED thickness at week 12 and BCVA at weeks 48 and 96; notably, patients with PED thickness ≥400 μm at week 12 lost vision over time, whereas patients with PED thickness <400 μm at week 12 gained vision over time. Further, the results of a retrospective analysis reported by Selvam et al^15^ suggest that PED composition is a biomarker of disease activity in eyes treated with anti-VEGFs. Various clinical practice evidence also supports PED as a biomarker of nAMD disease activity. Specifically, in an analysis of eyes treated with ranibizumab or aflibercept, Lai et al^16^ found that PED at baseline was a predictor of worse BCVA at 1 year. More recently, Veritti et al^33^ reported that there was a significant association between reduction in PED volume and improvement in BCVA among patients treated with faricimab over a 120-day period. In contrast to these reports, there is evidence to suggest that decreasing PED thickness/PED resolution may lead to geographic atrophy/loss of vision in some patients, particularly those with drusenoid PED.^34^, ^35^, ^36^ However, there is no evidence that decreasing PED thickness with faricimab leads to vision loss. As previously reported, vision outcomes in TENAYA/LUCERNE were similar between treatment arms through 2 years.^21^^,^^25^ Interestingly, results from the treatment agnostic TENAYA/LUCERNE analysis described herein indicated that eyes with persistent PEDs at the foveal center at week 12 tended to have less pronounced vision gains over time. Clinical practice evidence to date has also shown reductions in PED size coincide with stable visual acuity in patients treated with faricimab.^31^^,^^32^ In addition, findings from the HAWK/HARRIER trials showed no difference in BCVA outcomes between the brolucizumab and aflibercept 2 mg arms through 2 years,^37^ despite PED resolution being more pronounced with brolucizumab.^38^ Hence, overall, not flattening PED seems to be a greater threat to vision than flattening PED. Nevertheless, further research is needed to determine if there are certain patients with PED or specific PED characteristics who may be more likely to benefit from treatment than others. The impact of PED flattening with faricimab on vision also warrants evaluation over a longer period of time because 2 years is likely too short of a window to detect the full extent of atrophy that could be seen in an area of flattened PED.

The incidence of RPE tears was low in TENAYA/LUCERNE and consistent with published data for anti-VEGF agents from both clinical trials and clinical practice studies (0.5%–15.7%).^39^, ^40^, ^41^, ^42^, ^43^, ^44^, ^45^, ^46^, ^47^, ^48^

This post hoc analysis of the TENAYA/LUCERNE trials has several limitations, including the relatively small sample size for patients with serous PED and the lack of type 1 error (false positive) control in statistical testing. Given the latter, the results should be interpreted with caution and further confirmatory evidence is needed. Further, as maximum PED thickness was measured throughout the foveal scan (6-mm ETRDS grid), the point with maximum thickness may have varied between visits. We also acknowledge that caliper-based PED measurement may be limiting; therefore, follow-up algorithm-based PED volumetric analyses are planned. Finally, further investigation into the clinical significance of the differences in PED thickness reduction between faricimab and aflibercept is required.

In summary, we found that dual Ang-2/VEGF-A pathway inhibition with faricimab resulted in greater improvement in PED outcomes versus VEGF pathway inhibition alone with aflibercept in the head-to-head phase of TENAYA and LUCERNE. These findings are consistent with the greater and faster drying of retinal fluid seen over the same time period in these trials, as evidenced by retinal biomarker findings (CST, IRF, and SRF), and may underlie the rapid treatment interval extension observed with dual Ang-2/VEGF-A inhibition in TENAYA/LUCERNE. These results provide important information on the treatment effects on PEDs in clinical trials; clinical practice data will continue to be collected to further understand the clinical implications for patients with nAMD.
