# Supplementary material for: Reduction in Pigment Epithelial Detachment Thickness with Faricimab versus Aflibercept 2 mg during Head-to-Head Dosing in TENAYA/LUCERNE
Source: Ophthalmol Sci. 2026 Mar 10;6(5):101148. doi: 10.1016/j.xops.2026.101148 (PMC13084404; doi:10.1016/j.xops.2026.101148)

Figure S2. Reduction in maximum PED thickness with faricimab versus aflibercept in the head-to-head dosing phase in patients with presence of (A) any PED at baseline and (B) fibrovascular PED at baseline. *Nominal *P* < 0.05 versus aflibercept; **Nominal *P* < 0.01 versus aflibercept. *P* values are nominal and not adjusted for multiplicity; no formal statistical conclusion should be made based on the *P* values. [a]Within the 6-mm ETDRS grid. PED thickness results are based on a mixed model for repeated measures analysis on all patients randomized in the trials grouped according to the treatment assigned at randomization. The model adjusted for treatment group, visit, visit-by-treatment group interaction, baseline PED (continuous), PED type at baseline (fibrovascular vs. serous), baseline BCVA (≥ 74, 73–55, and ≤ 54 letters), baseline LLD (< 33 and ≥ 33 letters), region (United States and Canada, Asia, and the rest of the world), reading center (Vienna vs. Duke), and study (TENAYA vs. LUCERNE). Treatment policy strategy and hypothetical strategy were applied to non–COVID-19–related and COVID-19–related intercurrent events, respectively. 95% CIs are shown. Presence of PED defined as measured maximum thickness of PED within the 6-mm ETDRS grid at baseline. BCVA = best-corrected visual acuity; CI = confidence interval; COVID-19 = coronavirus disease 2019; ETDRS = Early Treatment Diabetic Retinopathy Study; LLD = low-luminance deficit; PED = pigment epithelial detachment.

A

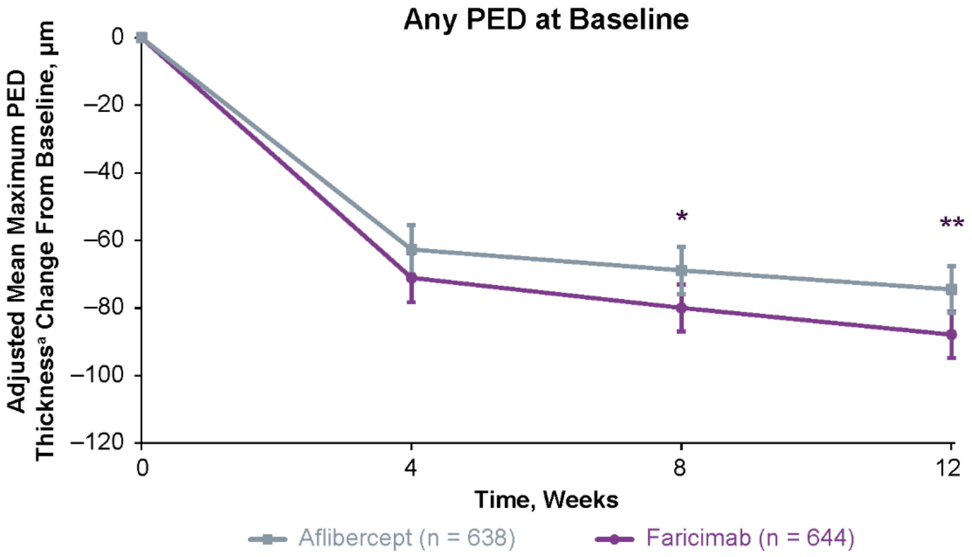

B

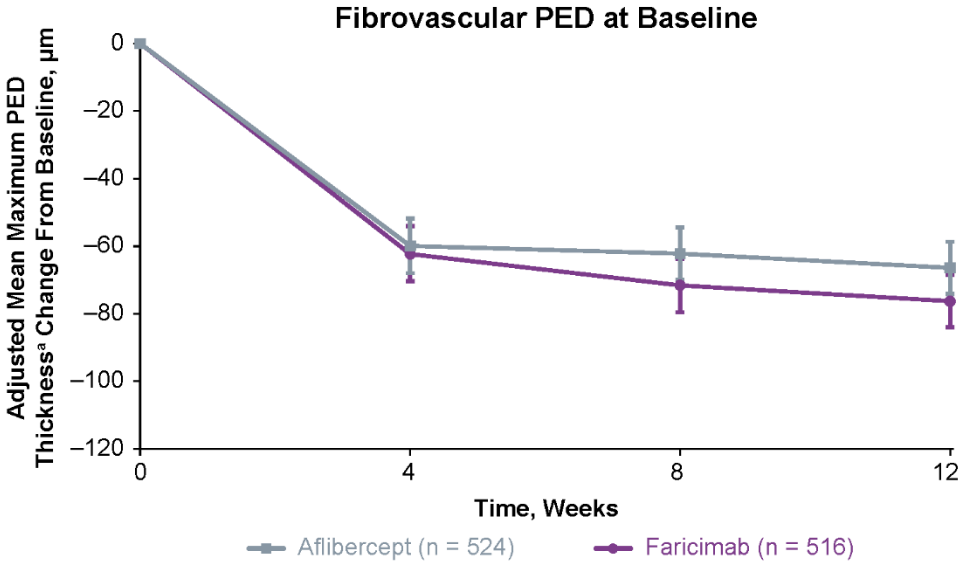

Supplement: Figure S2 [file mmc1.pdf]
