# Supplementary material for: Reduction in Pigment Epithelial Detachment Thickness with Faricimab versus Aflibercept 2 mg during Head-to-Head Dosing in TENAYA/LUCERNE
Source: Ophthalmol Sci. 2026 Mar 10;6(5):101148. doi: 10.1016/j.xops.2026.101148 (PMC13084404; doi:10.1016/j.xops.2026.101148)
Supplement: Figure S3 [file mmc2.pdf]

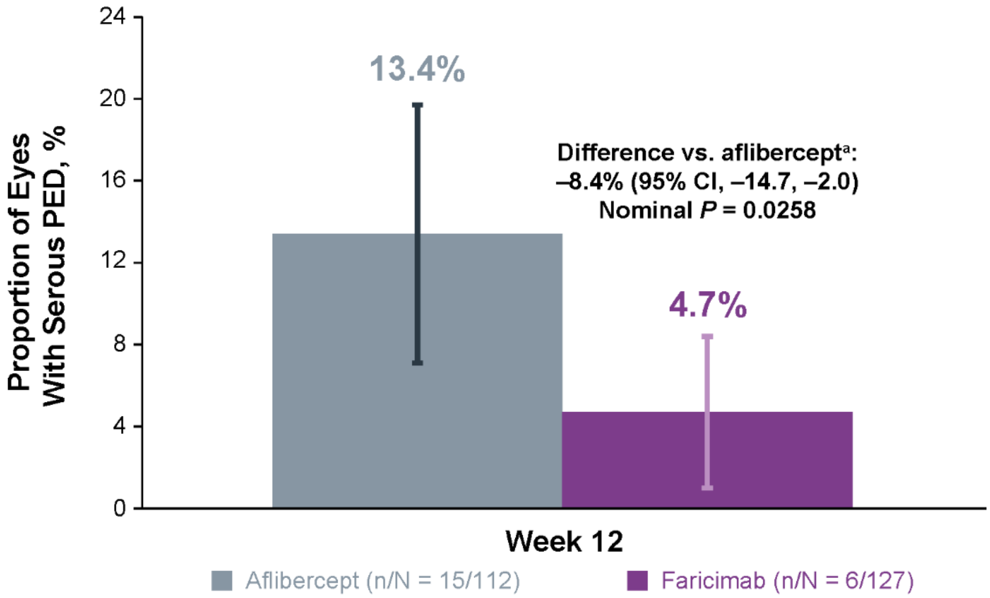

Figure S3. Proportion of eyes with serous PED baseline that continued to have serous PED at week 12 with faricimab versus aflibercept. Patients had serous PED at baseline. Predominantly serous and serous only PEDs are reported as serous PED. [a]Estimates are based on CMH test stratified by baseline BCVA (≥ 74, 73–55, and ≤ 54 letters), baseline LLD (< 33 and ≥ 33 letters), region (United States and Canada, Asia, and the rest of the world), and study (TENAYA vs. LUCERNE). Nominal *P* value is obtained with CMH test for superiority. *P* values are nominal and not adjusted for multiplicity; no formal statistical conclusion should be made based on the *P* values. 95% CIs are shown. BCVA = best-corrected visual acuity; CI = confidence interval; CMH = Cochran-Mantel-Haenszel; LLD = low-luminance deficit; PED = pigment epithelial detachment.
