# Supplementary material for: Reduction in Pigment Epithelial Detachment Thickness with Faricimab versus Aflibercept 2 mg during Head-to-Head Dosing in TENAYA/LUCERNE
Source: Ophthalmol Sci. 2026 Mar 10;6(5):101148. doi: 10.1016/j.xops.2026.101148 (PMC13084404; doi:10.1016/j.xops.2026.101148)

Figure S6. Treatment agnostic adjusted mean BCVA change from baseline at weeks 28, 48, and 112 in eyes with and without clinically relevant PED involving the foveal center at baseline. Clinically relevant PED was defined as presence of PED at the foveal center and maximum PED thickness ≥ 125 μm at baseline. * Nominal $P < 0.05$ versus eyes with clinically relevant PED involving the foveal center at baseline. *P* values are nominal and not adjusted for multiplicity; no formal statistical conclusion should be made based on the *P* values. BCVA results are based on a mixed model for repeated measures analysis. The model adjusted for baseline maximum PED thickness at foveal center group, visit, visit-by-maximum PED thickness at foveal center group interaction, baseline BCVA (continuous), sex, PED subtype at baseline, total lesion size (mm^2) at baseline, baseline central subfield thickness (internal limiting membrane to Bruch's membrane), choroidal neovascularization location by fundus fluorescein angiography at baseline and study (TENAYA vs LUCERNE). An unstructured covariance structure was used. Treatment policy strategy and hypothetical strategy were applied to non–COVID-19–related and COVID-19–related intercurrent events, respectively. 95% CIs are shown. BCVA = best-corrected visual acuity; CI = confidence interval; COVID-19 = coronavirus disease 2019; ETDRS = Early Treatment Diabetic Retinopathy Study; PED = pigment epithelial detachment.

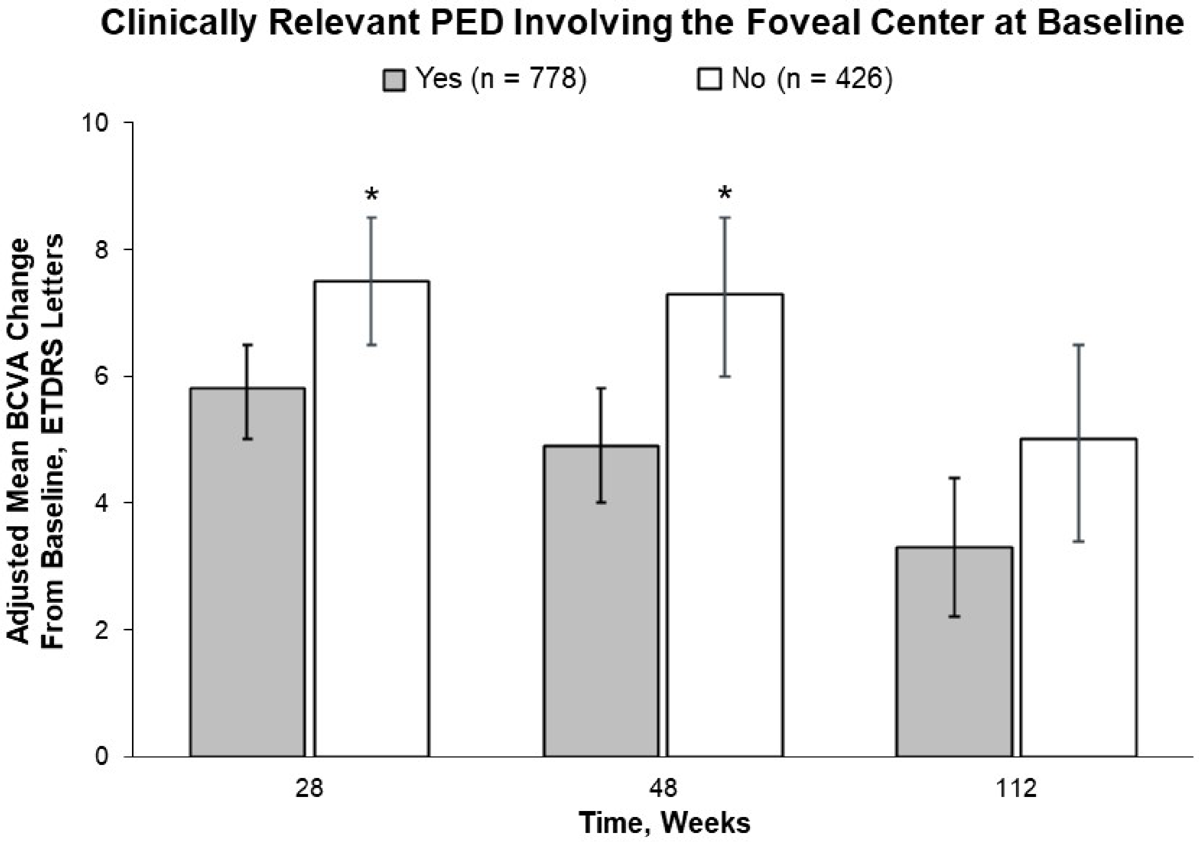

Supplement: Figure S6 [file mmc3.pdf]
