# Supplementary material for: Reduction in Pigment Epithelial Detachment Thickness with Faricimab versus Aflibercept 2 mg during Head-to-Head Dosing in TENAYA/LUCERNE
Source: Ophthalmol Sci. 2026 Mar 10;6(5):101148. doi: 10.1016/j.xops.2026.101148 (PMC13084404; doi:10.1016/j.xops.2026.101148)
Supplement: Table S2 [file mmc5.pdf]

Table S2. Baseline Ocular Characteristics for Study Eyes with Large, Serous, Fibrovascular PED at Baseline

Characteristic	Large PED (≥ 125 µm)		Serous PED		Fibrovascular PED	
	Faricimab up to Q16W (N = 500)	Aflibercept Q8W (N = 496)	Faricimab up to Q16W (N = 130)	Aflibercept Q8W (N = 114)	Faricimab up to Q16W (N = 517)	Aflibercept Q8W (N = 524)
BCVA (ETDRS letters), mean ± SD	60.2 ± 13.6	60.4 ± 12.9	62.2 ± 12.7	60.7 ± 12.3	59.5 ± 13.4	60.1 ± 13.3
BCVA category, n (%)						
≥ 74 (20/32 or better)	72 (14.4)	67 (13.5)	21 (16.2)	13 (11.4)	68 (13.2)	74 (14.1)
73−55 (20/40–20/80)	288 (57.6)	291 (58.7)	77 (59.2)	70 (61.4)	296 (57.3)	298 (56.9)
≤ 54 (20/80 or worse)	140 (28.0)	138 (27.8)	32 (24.6)	31 (27.2)	153 (29.6)	152 (29.0)
CST (µm),[a] mean ± SD	352.1 ± 117.3	355.5 ± 114.1	352.5 ± 112.9	361.2 ± 104.2	357.2 ± 122.2	356.1 ± 122.9
Intraocular pressure (mmHg), mean ± SD	15.0 ± 3.0	14.8 ± 3.0	15.0 ± 3.0	14.6 ± 2.6	14.9 ± 3.0	14.9 ± 3.0
Time since nAMD diagnosis (mos), n (%)						
≤ 1 mo	348 (69.6)	327 (65.9)	97 (74.6)	81 (71.1)	358 (69.2)	358 (68.3)
> 1 mo	122 (24.4)	147 (26.9)	24 (18.5)	27 (23.7)	134 (25.9)	147 (28.1)
Phakic, n (%)	284 (56.8)	278 (56.0)	70 (53.8)	70 (61.4)	302 (58.4)	284 (54.2)
Presence of IRF, n (%)	217 (43.4)	231 (46.6)	60 (46.2)	57 (50.0)	221 (42.7)	243 (46.4)
Presence of SRF, n (%)	326 (65.2)	331 (66.7)	80 (61.5)	78 (68.4)	349 (67.5)	354 (67.6)
CNV location type by FFA, n (%)						
Subfoveal	309 (61.8)	290 (58.5)	84 (64.6)	54 (47.4)	315 (60.9)	312 (59.5)
Juxtafoveal	120 (24.0)	121 (24.4)	29 (22.3)	33 (28.9)	126 (24.4)	131 (25.0)
Extrafoveal	64 (12.8)	76 (15.3)	16 (12.3)	26 (22.8)	66 (12.8)	67 (12.8)
CNV lesion type by FFA, n (%)						
Occult	293 (58.6)	253 (51.0)	91 (70.0)	61 (53.5)	251 (48.5)	246 (46.9)
Classic	109 (21.8)	112 (22.6)	12 (9.2)	11 (9.6)	164 (31.7)	158 (30.2)
Minimally classic	45 (9.0)	51 (10.3)	10 (7.7)	15 (13.2)	49 (9.5)	46 (8.8)
RAP	22 (4.4)	36 (7.3)	10 (7.7)	16 (14.0)	16 (3.1)	24 (4.6)
Predominantly classic	14 (2.8)	22 (4.4)	3 (2.3)	4 (3.5)	19 (3.7)	28 (5.3)
Total area of CNV lesion by FFA (mm^2), mean ± SD	5.1 ± 5.0	4.7 ± 4.3	5.5 ± 4.8	5.2 ± 4.6	4.5 ± 4.8	4.3 ± 4.1

BCVA = best-corrected visual acuity; CNV = choroidal neovascularization; CST = central subfield thickness; ETDRS = Early Treatment Diabetic Retinopathy Study; FFA = fundus fluorescein angiography; ILM = internal limiting membrane; IRF = intraretinal fluid; nAMD = neovascular age-related macular degeneration; PED = pigment epithelial detachment; Q8W = every 8 weeks; Q16W = every 16 weeks; RAP = retinal angiomatous proliferation; RPE = retinal pigment epithelium; SD = standard deviation; SRF = subretinal fluid.
This table includes all patients randomized in the trials grouped according to the treatment assigned at randomization.

[a]CST is measured as the distance between the ILM and RPE.
